# Human bone tissue-derived ECM hydrogels: Controlling physicochemical, biochemical, and biological properties through processing parameters

**DOI:** 10.1016/j.bioactmat.2024.09.007

**Published:** 2024-09-23

**Authors:** Yang-Hee Kim, Gianluca Cidonio, Janos M. Kanczler, Richard OC. Oreffo, Jonathan I. Dawson

**Affiliations:** a Bone and Joint Research Group, Centre for Human Development, Stem Cells and Regeneration, Institute of Developmental Sciences, University of Southampton, SO16 6YD, United Kingdom; b Department of Mechanical and Aerospace Engineering (DIMA), Sapienza University of Rome, Via Eudossiana 18, 00184, Rome, Italy; c Center for Life Nano- and Neuro-Science (CLN2S), Istituto Italiano di Tecnologia, Viale Regina Elena 291, 00161, Rome, Italy

**Keywords:** Hydrogels, Human bone, Extracellular matrix, Demineralization, Decellularization

## Abstract

Decellularized tissues offer significant potential as biological materials for tissue regeneration given their ability to preserve the complex compositions and architecture of the native extracellular matrix (ECM). However, the evaluation and derivation of decellularized matrices from human bone tissue remains largely unexplored. We examined how the physiochemical and biological properties of ECM hydrogels derived from human bone ECM could be controlled by manipulating bone powder size (45–250 μm, 250–1000 μm, and 1000–2000 μm) and ECM composition through modulation of enzyme digestion time (3-5-7 days).

A reduction in material bone powder size and an increase in ECM digestion time produced enhanced protein concentrations in the ECM hydrogels, accompanied by the presence of a diverse array of proteins and improved gelation strength. Human bone marrow-derived stromal cells (HBMSCs) cultured on ECM hydrogels from 45 to 250 μm bone powder, over 7 days, demonstrated enhanced osteogenic differentiation compared to hydrogels derived from larger bone powders and collagen gels confirming the potential of the hydrogels as biologically active materials for bone regeneration. Digestion time and bone powder size modulation enabled the generation of hydrogels with enhanced release of ECM proteins and appropriate gelation and rheological properties, offering new opportunities for application in bone repair.

## Highlights

• Human bone tissue-derived ECM hydrogels were successfully fabricated.
• Bone powder size and digestion time significantly affect the gelation speed, rheological strength, and degradation of ECM hydrogels.
• Smaller bone powder sizes and longer digestion times led to a higher concentration of diverse proteins in the ECM hydrogels.
• Human bone ECM hydrogels enhanced the behavior and function of bone marrow-derived stromal cells.

## Graphical abstract

**Figure:**
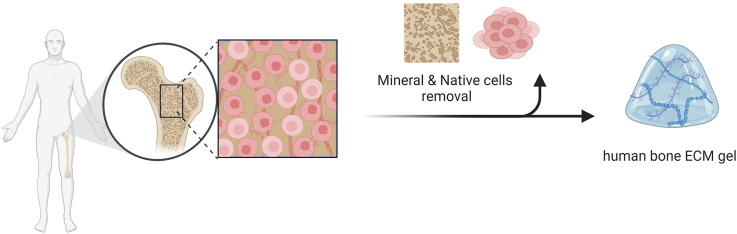

## 1 Introduction

Tissue engineering offers significant promise for the repair of damaged tissues harnessing cells, bioactive molecules, and biomaterials. In particular, biomaterials play a crucial role providing a microenvironment and, typically, delivery of molecules that can promote the functions of the requisite cells within the regenerating tissue. Various strategies have focused on developing and designing biomaterials that can mimic the structure and composition of the native extracellular matrix (ECM) [1,2]. Natural polymer-based hydrogels, such as collagen, alginate, chitosan, hyaluronic acid, and gelatin, display similar components as ECM and, in addition, generate fibrous and porous microstructures, regulating cell behavior including attachment, proliferation, and differentiation [3,4]. However, reproducing the complex mixture of structural and functional proteins in ECM, including the glycosaminoglycans (GAGs), collagens, proteoglycans, and growth factors, remains, to date, challenging.

Decellularization of tissues has emerged as a promising approach for the generation and subsequent application of biological materials. Following the removal of the cellular components from native tissues, the complex compositions and architecture of the ECM can be retained [5,6]. Furthermore, decellularization can reduce immunogenicity limiting associated issues around inflammatory responses and disease transmission. Indeed, decellularized ECM has been shown to advance cell behavior and constructive tissue remodeling responses [[7], [8], [9]]. To date, animal decellularized matrices have found application in various clinical products, including three-dimension implantable scaffolds, 2D sheets, and powders [6,10]. Recently decellularized ECMs have been formulated as hydrogels [11]. ECM hydrogels are primarily composed of proteins solubilized or digested from decellularized ECM with the capacity to form a gel at physiological temperature and pH. As a consequence, such hydrogels can be applied at a defect site through injection providing a more homogenous concentration of material in contrast to the use of ECM powders [12]. The most commonly used method for generating an ECM hydrogel remains the pepsin-mediated solubilization of decellularized tissue-derived powders. Indeed, pepsin enzyme can solubilize a range of proteins present within ECM and a number of studies have reported that ECM protein-based hydrogels retain a degree of bioactivity leading to improvements in cell proliferation, differentiation, and tissue repair [13,14] and, naturally, implementation in tissue engineering and regenerative medicine [5,11].

To date, there have been a limited number of studies examining decellularized ECM hydrogels derived from bovine and porcine bone tissues for bone tissue regeneration. These hydrogels have demonstrated the ability to enhance cell function and promote bone regeneration [15,16]. The current study examined the potential to generate human ECM gels from donor human bone graft material as an alternative to animal tissue-derived ECM hydrogels. Regardless of the tissue source, ECM hydrogels comprise natural ECM-based materials with low immunogenicity achieved by removing cellular components. While human tissue/organs are not readily available, they possess the least immunogenicity, making allotransplantation the gold standard of treatment. Animal tissue-derived ECM may elicit an immune response due to certain animal protein constituents, such as bovine collagen, proteoglycan link protein, glycan antigens, porcine endogenous retroviruses genes, and α-gal epitopes [[17], [18], [19], [20]].

In the preparation of porcine bone-derived ECM hydrogels, demineralized and decellularized bone powders (<40 μm) were treated in pepsin solution for 1 h [21]. Similar, ECM hydrogels derived from bovine bone powders were fabricated after 96 h of digestion in a pepsin solution [15]. However, to date, the effect of tissue size and digestion time on protein concentration, gelation, rheological and biological properties of ECM hydrogels remain unknown. Critically, there is no study on the fabrication of hydrogels derived from human bone tissue ECM focusing on fabrication parameters. This is crucial for minimizing the batch-to-batch variation, especially given the wide range of bone powder sizes, and maximizing the regenerative functions of the ECM hydrogels by optimizing the proportions of ECM proteins. Our study focuses specifically on hydrogels derived from the extracellular matrix of demineralized and decellularised human bone powders, which differs from conventional demineralized bone powders.

Therefore, the current study has examined the influence of particle size and digestion time - two key parameters involved in generating ECM hydrogels from demineralized and decellularized human bone - on the physicochemical and biological properties of human bone ECM hydrogels and their potential application in bone tissue repair. Human trabecular bone tissue was obtained from human femoral heads following orthopedic elective surgery and demineralized human bone powders of varying sizes (45–250 μm, 250–1000 μm, and 1000–2000 μm) were prepared. The powders were subjected to pepsin treatment for 3, 5, or 7 days. We evaluated the gelation and rheological properties of the resulting ECM hydrogels based on powder size and digestion time. Hydrogel microstructure was examined using scanning electron microscopy and micro-computed tomography. In addition, the efficiency of protein digestion was assessed by measuring cellular and protein concentrations. The proteomics profile of the derived hydrogels was evaluated using mass spectrometry. Finally, human bone marrow-derived stromal stem cell attachment, spreading, migration, proliferation, and differentiation to determine biological functionality, were examined on the human ECM hydrogels.

**Fig. 1:**
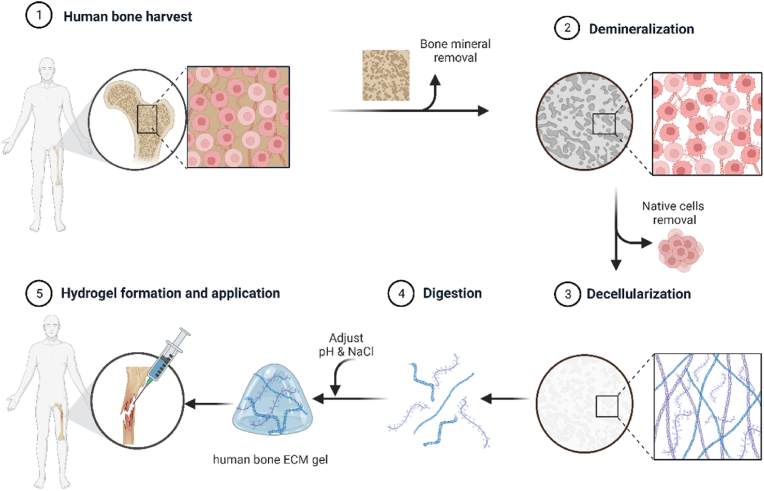
**Schematic illustration of ECM hydrogel derived from demineralized and decellularized human bone.**Human trabecular bones were demineralized and decellularised. The digested extracellular matrix proteins were crosslinked, forming a gel to apply to a defect.

In summary, this study set out to examine the critical impact of powder size and digestion time on the properties of ECM hydrogels derived from human bone tissue, to offer insights on the ECM physicochemical properties for the development of effective biomaterials for bone repair applications (Fig. 1).

## 2 Materials and methods

### 2.1 Preparation of human bone tissue-derived ECM hydrogels

#### 2.1.1 Demineralization of human trabecular bone

**Fig. 2:**
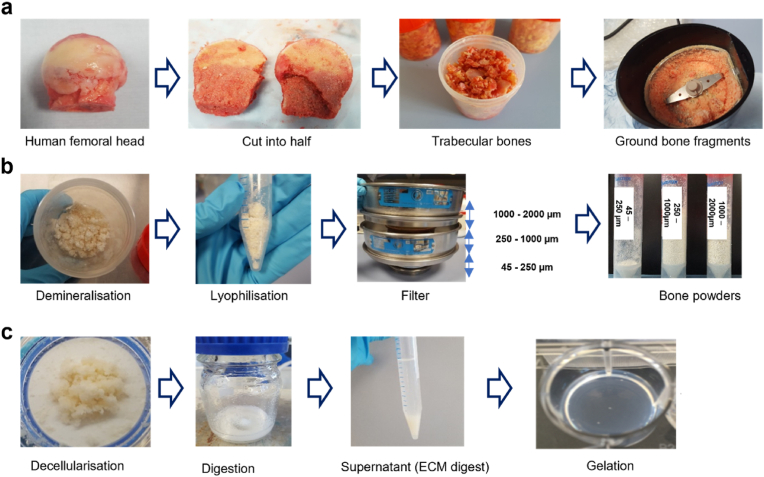
**Preparation process of ECM hydrogel derived from demineralized and decellularized human bone.** (a) Human femoral heads from haematologically normal patients, both female and male, aged 65 ± 12 years old, were cut in half, and the trabecular bones collected and fragmented. (b) The fragments underwent demineralization in 0.5N HCl for 24 h, followed by lyophilization. The lyophilised powders were then sieved through stainless steel sieves to obtain powders in the ranges of 45–250 μm, 250–1000 μm, and 1000–2000 μm. (c) After decellularization in a Trypsin/EDTA solution, the powders were digested in a pepsin solution for 3, 5, and 7 days, respectively. The supernatant from the digestion solution was mixed with 0.1N NaOH, 10 × PBS, and 1 × PBS and incubated at 37 °C for 1 h to induce gelation.

Human femoral heads were collected from haematologically normal patients undergoing elective hip arthroplasty. Only tissue samples that would have been discarded were used following informed consent from the patients in accordance with the Southampton & Southwest Hampshire Local Research Ethics Committee (Ref: 194/99/w). The femoral heads from female and male patents, aged 65 ± 12 years old were cut using an isomet low speed precision cutter (Buehler LTD. US), and trabecular bones from the femoral heads were collected using a bone nipper (Fig. 2a). The collected trabecular bones were frozen in liquid nitrogen and ground in a commercial coffee grinder. To remove residual fats from the bones, the ground bones in 50 ml tubes were washed with phosphate-buffered saline (PBS, Lonza, Slough, UK) containing 2 % penicillin/streptomycin (P/S, Sigma–Aldrich, Poole, UK) until the tubes were no longer smeared with fat.

Ground human trabecular bones were demineralized using a modification of previously reported methods [15] (Fig. 2b). The bone fragments were treated in a 0.5 N HCl (Fisher Scientific) at room temperature for 24 h. To solubilize the lipids in the bone, the fragments were added to a 1:1 mixture of chloroform and methanol for 1 h, followed by rinsing in methanol and then deionized water several times. After lyophilization (Lablyo, Wolflab, UK), the demineralized bone matrix (DBM) fragments were ground using a homogenizer (T10 basic Ultra-turrax, IKA LTD, Oxford, UK). The powders went passed through stainless steel sieves with 45, 250, 1000, and 2000 μm pore size (Fisher Scientific) to obtain the DBM powders with 45–250 μm, 250–1000 μm, and 1000–2000 μm sizes.

#### 2.1.2 Decellularization and digestion

The DBM powders were decellularized in a solution of 0.05 % trypsin with 0.02 % ethylenediaminetetraacetic acid (trypsin/EDTA, Lonza) at 37 °C and 5 % CO_2_ incubator under agitation for 24 h (Fig. 2c). The powders were collected using cell strainer (40 μm, Fisher Scientific) and rinsed in PBS twice and in deionized water twice, followed by overnight freeze-drying. Pepsin (Sigma–Aldrich) was dissolved in 0.01N HCl (1 mg/ml). The demineralized and decellularized bone powders with various sizes (45–250 μm, 250–1000 μm, 1000–2000 μm) were separately added to the pepsin solution and enzymatically digested under agitation for 3, 5, and 7 days, respectively, at room temperature (Table 1). The final concentration of the bone powders in the pepsin solution was 20 mg/ml, and the solutions were then centrifuged at 2000 rpm at 15 min to obtain supernatants referred to as ECM digests. The supernatants were collected and stored at −20 °C until required.

**Table 1:** Changes of digestion time and powder size for optimizing bone ECM hydrogels.

Size (μm) Digestion time	45–250	250–1000	1000–2000
3 days	45–D3	250–D3	1000–D3
5 days	45–D5	250–D5	1000–D5
7 days	45–D7	250–D7	1000–D7

#### 2.1.3 Gelation of human bone ECM hydrogels

The ECM hydrogels were induced by neutralizing the pH and salt concentration of the ECM digests, followed by incubation at 37 °C, as previously described [15,22]. Briefly, ECM digests were mixed with one-tenth of the digest volume of 0.1 N NaOH, one-ninth of the digest volume of 10 × PBS, and then diluted with 1 × PBS (referred to as ECM gelation buffer) on ice to create a 16 mg/ml ECM gel. Subsequently, the neutralized pre-gel solution was incubated for 1 h at 37 °C.

### 2.2 Gelation characterization

#### 2.2.1 Tube inversion and turbidimetric studies

The gelation property of human bone ECM hydrogels was determined through tube inversion and turbidimetric gelation assays. For the tube inversion study, the neutralized pre-gel solutions (16 mg/ml) obtained from various bone ECM powder sizes and digestion times were placed in 1.5 ml of Eppendorf tubes and the volume of each solution in the tubes was marked. After inverting the tubes, images were taken using a digital camera. Subsequently, the tubes were incubated at 37 °C for 1 h, inverted again, and images were taken.

Next, turbidimetric gelation kinetics of the ECM hydrogels were evaluated spectrophotometrically as previously described [22,23]. The neutralized pre-gel solutions were prepared on ice, and 100 μl of the solution was transferred to a pre-cooled 96 well-plate. The plates were placed in a pre-warmed microplate reader at 37 °C, and the turbidity of each well was measured at a wavelength of 450 nm every 2 min for an hour (n = 4). The absorbance values for each well were recorded and averaged.

Equation (1) $$NA=\frac{A\;-A_{0}}{A_{\max}\;-A_{0}\;}$$

These readings were then scaled from 0 (at time 0) to 100 % (at maximum absorbance) for normalizing absorbance (NA), as shown in Equation (1), where A is the absorbance at a given time, A0 is the initial absorbance, and A_max_ is the maximum absorbance. The time needed to reach 50 % of the maximum turbidity measurement (e.g., maximum absorbance value) was defined as t_1/2_ and the lag time (t_lag_) was calculated by the intercept of the linear region of the gelation curve with 0 % absorbance. The speed (S) of the gelation based on turbidimetric measurements was determined by calculating the maximum slope of the growth portion of the curve as previously described [22].

#### 2.2.2 Rheological characterization

The rheological characteristics of human bone ECM hydrogels were determined using a rheometer (MCR 92, Anton Paar, UK). 1 ml of pre-gel solution was loaded onto a pre-warmed (37 °C) 25 mm parallel plate and incubated at 37 °C for 1 h under humanify condition. An amplitude sweep experiment was conducted for the human bone ECM at 0.01–200 % stain at a constant angular frequency of 1 rad/s.

#### 2.2.3 Fourier transform infrared spectroscopy (FTIR) analysis

The neutralized pre-gel ECM and collagen solutions (with ECM buffer), ECM digest, and collagen (without ECM buffer) were analyzed by FTIR (FT-IR-4700, Jasco, Maryland, USA). 16 mg/ml of ECM digest and ECM pre-gel solution and ECM digest, and 300 μg/ml type I collagen and collagen solution (buffer) were drop casted and dried overnight. The detection range of the spectrometer was 399–4000 cm^−1^, and the data were measured with an interval of 0.96 cm–1 at room temperature. The raw FT-IR data was processed with a spectrum software (Spectra Manager™ Suite, Jasco) for smoothing and baseline correction.

*Microstructure observation*: Microstructure of human bone ECM hydrogels were examined using scanning electron microscopy (SEM). The pre-gel ECM solutions were neutralized and incubated at 37 °C for 1 h, followed by freeze drying to dehydrate the gels. The dried gels were coated with platinum and analyzed using an SEM (FEI Quanta 200) at accelerating voltages of 5 kV. Images were captured at magnifications of 55×, 200 × , 1000 × and 4000 × . The area of pores was measured using ImageJ software (n = 3).

Additionally, the freeze-dried ECM hydrogels contained in microcentrifuge tubes were individually scanned using Micro-Computed Tomography (μ-CT, SkyScan 1176 (Bruker, Kontich, Belgium). The scanning parameters were set to an X-ray source voltage of 50 kV, current of 500 μA, exposure time of 496 ms, and a voxel size of 18 μm.

To observe the structure of the hydrogels and the presence of various proteins derived from human bone, histological staining was performed. The ECM hydrogels were embedded in optimal cutting temperature compound (OCT, Fisher Healthcare™ Tissue-Plus™) and frozen at −20 °C. Sections in thickness of 8 μm were obtained using a Cryostat (CM 1850, Leica Biosystems) and mounted with super cryo-mounting medium type R3 (SCMM R3, Section lab Co. Ltd, Japan). The sections were then stained using Alcian Blue/Sirius Red staining, including haematoxylin. Images were obtained using a microscopy (Axiovert 200, Zeiss).

### 2.3 Quantification of cellular, protein, growth factor contents

The cellular content of the various ECM digests was determined using a Quant-iTTM PicoGreen dsDNA assay kit (Invitrogen, Fisher Scientific, Paisley, UK). Following the manufacturers’ protocol, 100 μl of ECM digest samples and DNA standards ranging from 0 to 1000 ng/ml were added to each well of a 96 well plate and mixed with 100 μl of the working solution (n = 4). The plate was covered with foil and incubated at room temperature for 5 min. The sample fluorescence was measured at excitation and emission wavelengths of 475 nm and 550 nm, respectively, using a microplate reader (Glomax, Promega, Southampton, UK). Based on a standard curve, the concentration of DNA in the ECM digests was determined.

The total protein concentration of the ECM digests was measured using a DC protein assay kit (Bio-Rad, Watford, UK) following the manufacturer's instruction. Briefly, 5 μl of protein (bovine serum albumin) standard and the digest samples were added to each well followed by addition of 225 μl of the working reagent (n = 4). After 15 min, the absorbance was read at 750 nm using the microplate reader.

The collagen and sulfated glycosaminoglycan (sGAG) concentrations of the digests were also quantified using the Sircol collagen assay and BlyscanTM Sulfated Glycosaminoglycan assay (Biocolor Lt, Carrickfergus, UK). For the collagen assay, the digests were diluted with a pepsin solution, and dye reagents were added to wells containing the diluted ECM digests and standard samples. After washing the dye-labeled collagen, dye release reagent was added to each sample, followed by 30 min of agitation (700 rpm). The absorbance was read at 560 nm using the microplate reader. To measure the concentration of sGAG in the ECM digest, the ECM digest samples were mixed with Blyscan dye in a gentle mechanical shaker for 30 min. After removing non-sGAG dye, 500 μl of dissociation reagent was added to each sample, followed by 10 min of incubation to release and recover sGAG-bound dye. Each sample was then transferred to a 96-well plate, and absorbance was read at 650 nm. The concentration of collagen and sGAG in the ECM digests was determined based on their standard curves.

The quantification of bone morphogenetic growth factor (BMP)-2 and vascular endothelial growth factor (VEGF) content was conducted using human BMP-2 and VEGF Quantikine enzyme-linked immunosorbent assay (ELISA) Kits (R&D, Bio-Techne, Abingdon, UK), respectively. Those assays were performed according to the manufacturer's instruction (n = 3) and the concentration of growth factors in the ECM digests was determined based on their standard curves.

The content of all the components per dry weight were calculated based on the average weights of dried ECM hydrogels.

### 2.4 Proteomic analysis

#### 2.4.1 Sample preparation

ECM digests derived from various bone powder sizes and treated digestion times were prepared for mass spectrometry. Briefly, ECM digests were solubilized in 5 M urea with 50 mM of (NH_4_)HCO_3_ and vortex for 30 s. To reduce disulfide bonds, 5 mM dithiothreitol was added to the samples, followed by 1 h incubation under agitation (1000 rpm) at 57 °C. Free cysteines were alkylated by adding iodoacetamide at a final concentration of 8.3 mM to the samples for 20 min in the dark. 100 μl of the samples were mixed with 50 mM Tris buffer, and then pre-digested with Trypsin/Lys-C Mix (Mass spec (MS) grad, Promega). After overnight incubation at 37 °C in the dark, the samples were acidified with trifluoroacetic acid (TFA, 1 % v/v). A 96 well sorbent plate (Oasis PRIME, HLB plate, Water™) was equilibrated with 100 % MS grade acetonitrile (ACN), and then washed with 0.1 % TFA in 99/1H_2_O/ACN. The samples were added to each well and desalted peptides with 0.1 % TFA.

#### 2.4.2 Liquid chromatography-tandem mass spectrometry (LC-MS/MS)

Peptides were reconstituted in 0.1 % formic acid and applied to an Orbitrap Fusion Tribrid Mass Spectrometer with a nano-electrospray ion source as previously described. Peptides were eluted with a linear gradient of 3–8% buffer B (Acetonitrile and 0.1 % Formic acid) at a flow rate of 300 nl/min over 5 min and then from 8 to 30 % over a further 192 min. Full scans were acquired in the Orbitrap analyzer using the Top Speed data dependent mode, preforming a MS scan every 3 s cycle, followed by higher energy collision-induced dissociation (HCD) MS/MS scans. MS spectra were acquired at resolution of 120,000 at 300–1500 *m*/*z*, RF lens 60 % and an automatic gain control (AGC) ion target value of 4.0e5 for a maximum of 100 ms and an exclusion duration of 40s. MS/MS data were collected in the Ion trap using a fixed collision energy of 32 % with a first mass of 110 and AGC ion target of 5.0e3 for a maximum of 100 ms.

Raw data files were analyzed using Peaks Studio 10.0 build 20190129. Relative protein quantification was performed using Peaks software and normalized between samples using a histone ruler [24]. The mass spectrometry proteomics data have been deposited to the ProteomeXchange Consortium via the PRIDE [25].

### 2.5 In vitro characterization

#### 2.5.1 HBMSCs cell culture

Human bone marrow stromal cells (HBMSCs) were isolated from the human femoral bone marrow obtained from Southampton General Hospital with the approval of the local ethics committee (LREC194/99/1). The HBMSCs were isolated by repeat perfusion of the marrows and filtration through 70 μm filter before centrifugation at 1200 rpm for 5 min. The HBMSCs pellets were resuspended in minimum essential medium alpha modification (α-MEM) with supplements of 10 % fetal bovine serum (FBS) and 1 % penicillin/streptomycin (P/S), and culture expanded in monolayer at 37 °C and under humidified 5 % CO_2_. The media was changed every 3 days.

#### 2.5.2 Cell attachment, spreading and migration

For cell attachment study, 300 μl of pre-gel solution was added to a coverslip placed in a well in 24-well culture plate. The plate was then covered with parafilm to prevent solution evaporation and incubated for 1 h. After the incubation, HBMSCs were seeded at 1 × 10^4^ cells/well and incubated 30 min. They were then fixed with 2.5 % glutaraldehyde in PBS. After rinsing in deionized water, the samples were dehydrated using graded ethanol (50 %, 70 %, 90 %, 95 % and 100 %). The cells on the ECM hydrogels were observed using a SEM at 5 kV, and images were taken at magnifications of 55×, 10,000 × and 25,000 × .

For cell migration behavior evaluation, 100 μl of pre-gel solution was dropped onto the centre of a well in 12-well culture plate. The plate was then covered with parafilm to prevent the solution from evaporation and then incubated for 1 h. While waiting for gelation, the microscope chamber was set up at 37 °C and 5 % CO_2_. HBMSCs were seeded on the hydrogels in the 12-well culture plate at a density of 1 × 10^4^ cells/well, and the plate was placed on the microscope stage. Time-lapse images were recorded continuously at 6 min intervals for 24 h.

#### 2.5.3 Cell viability and proliferation

Cell viability was investigated after 1, 3 and 7 days of culture using confocal imaging following previously employed protocol [26,27]. Briefly, the hydrogels were washed twice with 1 × HBSS. Scaffolds were then incubated in a diluted serum-free culture media solution of Calcein AM (C3099, Invitrogen, Thermo Fisher Scientific, UK) at 37 °C and 5 % CO_2_ balanced air for 1 h, following the manufacturer's protocol. Living cells were stained by both Calcein AM and DiD (green and red). Cells non-metabolically active (dead) were not stained with Calcien AM (Green) but only with DiD (red). Scaffolds were imaged using a confocal scanning microscope (Leica TCS SP5, Leica Microsystems, Wetzlar, Germany). Image analysis was carried out using Image J with an automated script. Cell proliferation was calculated normalizing the number of viable cells with the volume of interest (VOI).

Cell proliferation evaluation was determined using the WST-1 colorimetric assay (Roche, Germany). ECM hydrogels were prepared in wells of 48 well plates, and HBMSCs were seeded at a density of 1 × 104 cells/well and cultured for 1, 3, and 7 days. At each time point, 200 μl of WST-1 working solution was added to each well and incubated for 1 h. The absorbance was measured at 450 nm using a microplate reader (Glomax, Promega, Southampton, UK) (n = 5).

#### 2.5.4 Osteogenic differentiation

For Alkaline phosphatase (ALP) staining, Collagen and ECM gel coated surfaces (2D), and collagen and ECM hydrogels (3D) samples were prepared in 24 well plate. HBMSCs were then seeded at a density of 2 × 10^4^ cells/well with and cultured with a culture medium containing 50 mM ascorbic acid, 10 μM dexamethasone, 10 μM vitamin D3. The differentiation medium was changed every 3 days. After 7 days of cell culture, the cells were fixed in ethanol, and applying 100 μl of 4 % (v/v) Naphthol AS-MX phosphate (Sigma) and 0.0024 % (w/v) fast violet B-salt (Sigma) mixed in deionized water. Cells were incubated at 37 °C for up to 60 min, when the reaction was stopped the images were captured and processed using a Zeiss Axiovert 200 microscope and Axiovision software V4.0. Cell Profiler software was used to calculate the relative ALP staining intensity.

Alkaline phosphatase activity was measured using a colorimetric assay (P-nitrophenol phosphate (pNPP) turnover) measuring absorbance at 410 nm on an ELx800 spectrophotometer. In brief, 10 μl of cell lysate was transferred to a 96-well clear assay plate and made up to 100 μl with 90 μl phosphatase substrate (Sigma) in 1.5 M alkaline buffer solution (Sigma). The cell lysate was incubated at 37 °C for up to 40 min and terminated with 100 μl of 1 M sodium hydroxide prior to reading on the spectrophotometer. ALP activity was normalized by DNA content (n = 6). The DNA was quantified by the picoGreen dsDNA Quantitation Assay (Invitrogen, Paisley, UK). This assay is an ultrasensitive fluorescent nucleic acid stain of double-stranded DNA. Plates were read on an FLx cytofluor microplate reader (BioTech, Winooski, USA), and fluorescence was observed using 480 nm excitation and 520 nm emission.

For quantitative RT-PCR analysis, total RNA was extracted and purified from HBMSCs that were cultured on collagen and ECM hydrogels using the Qiagen RNeasy Mini Kit. cDNA was synthesized using SuperScript VILO cDNA synthesis kit (Life Technologies, Gibco, Cambridge Bioscience). Quantitative PCR was performed using Power SYBR Green PCR Master Mix (Invitrogen Lift Technology). The reaction was made up with cDNA, SYBR Green and reverse and forward primers for the gene of interest (Table 2).

**Table 2:** Primer sequences used for quantitative RT-PCR analysis.

Gene name	Forward primer sequence (5′–3′)	Reverse primer sequence (5′–3′)	Amplicon length (bp)
GAPDH	GACAGTCAGCCGCATCTTCTT	TCCGTTGACTCCGACCTTCA	86
ALP	GGAACTCCTGACCCTTGACC	TCCTGTTCAGCTCGTACTGC	86
RUNX2	GTAGATGGACCTCGGGAACC	GAGGCGGTCAGAGAACAAAC	78
COL1A1	GAGTGCTGTCCCGTCTGC	TTTCTTGGTCGGTGGGTG	52
OCN	GGCAGCGAGGTAGTGAAGAG	CTCACACACCTCCCTCCTG	102

All reactions were performed on the 7500 real-time PCR system (Applied Biosystems, Förster, CA) (n = 4). The targeted genes expression levels were normalized to Glyceraldehyde 3-phosphate dehydrogenase (GAPDH) and were then calculated using the 2−ΔΔCt method with reference to the control group (TCP).

#### 2.5.5 Osteogenic differentiation

Calcium deposition and mineralization were assessed by alizarin red S staining. Collagen and ECM hydrogels were prepared in 24-well plates, and HBMSCs were seeded at a density of 1.2 × 10^4^ cells/well either on top of the hydrogel surface or inside the hydrogels. Cells were cultured with mineralization medium containing 50 μM ascorbate-2-phosphate, 10 nM dexamethasone, and 2 mM beta-glycerophosphate, and the medium was changed every 3 or 4 days. After 14 and 21 days, cells were washed with PBS twice and fixed with 4 % paraformaldehyde for 15 min at room temperature. After fixation, cells were stained with 40 mM Alizarin red S solution (pH 4.1–4.3, #A5533, Sigma-Aldrich) for 1 h at room temperature on a shaker. The staining solution was discarded, and the cells were washed with deionized water until the stain was no longer released. Images were captured with a stereomicroscope attached to a digital camera (Canon Powershot G2).

To observe the cross-section of staining in hydrogels, the samples were embedded in optimal cutting temperature compound and snap-frozen. The samples were sectioned with a thickness of 10 μm using a cryostat (Leica CM1850). After washing off the residue of OCT, images were captured using a Zeiss Axiovert 200 microscope (Carl Zeiss).

### 2.6 Statistical analysis

Statistical analysis was performed using GraphPad Prism 8 (GraphPad.

Software, La Jolla, CA) with a one-/two- ANOVA test and Tukey's multiple comparison test. The data are presented as mean ± standard deviation (SD). Values of p < 0.05 were considered statistically significant.

## 3 Results and discussion

There is significantly interest in replicating the properties of the native ECM in the fields of biomaterials and tissue regenerative medicine and the current study has developed hydrogels derived from human bone ECM for orthopedic reparative applications.

Herein, we tested the hypothesis that the size of ECM bone powder particles and the duration of ECM digestion are crucial in determining the quality and quantity of proteins derived from the ECM, consequently influencing the rheological and physicochemical properties of the resulting hydrogel. This study investigated the potential of human bone ECM hydrogels to promote bone tissue repair through examination of human stromal cell function (Fig. 2).

### 3.1 Bone powder size and digestion time affect gelation speed, rheological strength, and degradation of ECM hydrogels

**Fig. 3:**
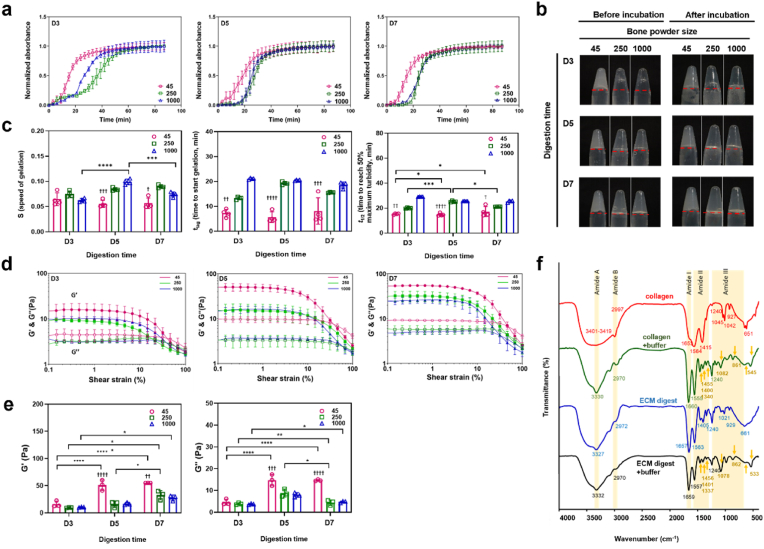
**Gelation and rheological characterizations of ECM hydrogels from various powder sizes and digestion time.** (a) Turbidimetric gelation kinetics of ECM hydrogels. pH-neutralized ECM pepsin digests were added to the wells of a pre-warmed 96-well plate (37 °C), and the absorbance at 405 nm was measured at 3-min intervals (n = 4). The values were normalized between 0 (the initial absorbance) and 1 (the maximum absorbance). (b) Photograph images of each gel after pH neutralization in inverted tubes before and after incubation at 37 °C. The red line indicates the initial volume of the gel before inversion. (c) S (speed of gelation), t_lag_ (time to start gelation), and t_1/2_ (time to reach 50 % maximum turbidity) were calculated based on turbidimetric curves. (d and e) Storage modulus (G′, open marks) and loss modulus (G″, closed marks) were monitored as hydrogels underwent an amplitude sweep of 0.1–100 % strain at a constant angular frequency. Data represent means ± standard deviation for n = 3. (f) Fourier transform infrared spectroscopy (FTIR) spectra of ECM digest compared with collagen with and without ECM buffer were analyzed. The spectrometer's detection range was 399–4000 cm-1, and the data were measured with an interval of 0.96 cm–1 at room temperature. Statistical significance was determined using a two-way ANOVA test with Tukey's multiple comparisons test (∗p < 0.05 ∗∗p < 0.005 ∗∗∗p < 0.0005∗∗∗∗). The † symbol indicates a significant difference within the groups at the same digestion time). Data represent mean ± SD, N = 4.

To investigate the influence of bone powder size and digestion time, on the gelation and rheological properties of ECM hydrogels, several types of human bone ECM hydrogels were prepared (Table 1) and turbidimetric gelation kinetics analysis undertaken. The gelation kinetics of all the human bone ECM hydrogels exhibited a sigmoidal pattern (Fig. 3a). Hydrogels derived from bone powder size ranging from 45 to 250 μm (45-D3/D5/D7), regardless of digestion time, demonstrated significantly sharper slopes and shorter t_lag_ and t_1/2_ compared to those derived from 250 to 1000 μm and 1000–2000 μm (Fig. 3c) bone powders (Fig. 3c). Specifically, the 45–D3/D5/D7 hydrogels displayed gelation approximately 7 ± 3 min after incubation at 37 °C, while the 250–D3/D5/D7 and 1000–D3/D5/D7 hydrogels exhibited gelatin times of 16 ± 0.4 and 20 ± 0.5 min, respectively.

For the gelation process, various ECM digests were mixed with the ECM gelation buffer, and the resulting pre-gel ECM solutions were placed in individual tubes and gently inverted. The pre-gel solutions for 250–D3, 250–D5, 1000–D3 and 1000–D5 immediately flowed down, while other samples remained stable in the tubes (Fig. 3b). After 1 h of incubation at 37 °C, except for the 250–D3 and 1000–D3 samples, all the ECM hydrogels were observed to maintain their structure in the inverted tubes.

We also evaluated the rheological characteristics of the hydrogels to determine the impact of bone powder size and digestion duration on the strength of human the bone ECM hydrogels (Fig. 3d and e). The storage (G’) moduli of the hydrogels derived from 45 to 250 μm bone powders, especially after 5 and 7 days of digestion, were significantly higher compared to those derived from 250 to 1000 μm and 1000–2000 μm bone powders. The highest storage modulus was observed in the 45-D7 hydrogels (55.0 ± 0.1 Pa), which was three-times higher than the modulus of the 45-D3 (16.90 ± 5.7 Pa) hydrogel. No significant difference was observed between the modulus of the 45-D7 and the 45-D5 (50.9 ± 9.6 Pa) hydrogels. In the case of ECM hydrogels derived from 250 to 1000 μm and 1000–2000 μm bone powders, there were no clear differences in storage and loss moduli was observed between D3 and D5 hydrogels. However, the moduli of the 250-D5 hydrogel were significantly higher than those of 1000-D5 hydrogel. These rheology results suggest that a digestion time of 5 days enabled solubilization of a variety of proteins from the ECM to generate stiff hydrogels using 45–250 μm bone powders. However, a greater time span was required to digest proteins from the large bone powders.

To test hydrogel stability and degradation *in vitro*, we evaluated the amount of released proteins from the ECM hydrogels following incubation in PBS for 24 h, followed by collagenase-PBS for an additional 24 h at 37 °C (Supplementary Fig. S1). Following 1 h of incubation in PBS, approximately 20–25 % of the proteins were released from the ECM hydrogels, and this percentage remained consistent even after 23 h. However, upon addition of the collagenase-PBS solution, released protein concentrations rapidly increased across all hydrogels following enzymatic stimulation by collagenase. Notably, ECM hydrogels derived from smaller bone powder samples exhibited a significantly slower release profile (Supplementary Fig. S1a), indicating that these hydrogels underwent degradation at a slower rate compared to the larger bone powder-derived hydrogels.

Among the 45-μm bone powder-sized ECM hydrogels, prolonged digestion times resulted in slower degradation, although this trend was not observed for the 250-μm and 1000-μm bone powder samples. This is likely because smaller bone powder ECM hydrogels contain significantly higher concentrations of proteins, including collagen (Fig. 5), which promotes strong and rapid gelation. This enhanced gelation leads to improved mechanical properties and, consequently, slower degradation of the hydrogels due to the increased crosslinking of proteins. These results indicate bone powder size and digestion times play a crucial role in determining the gelation and rheological properties of ECM hydrogels, as well as their degradation capacity.

### 3.2 The gelation mechanism of ECM hydrogels

As with the gelation of collagen extracts, gelation of human-based ECM hydrogels is induced by neutralizing the pH and salt concentration of the ECM digests, followed by incubation at 37 °C, as previously described [15,22]. To examine the mechanism of gelation and the contribution of collagen, we assessed the effect of the neutralizing ECM buffer on the chemical structures of type I collagen and ECM digest pre-gels using Fourier Transform Infrared Spectroscopy (FTIR) (Fig. 3f). The spectra of all samples examined exhibited distinct patterns across six regions; 3400-3420 cm^−1^ (amide A), 3000-2970 cm-1 (amide B), 1660-1657 cm^−1^ (amide I), 1557-1563 cm^−1^ (amide II), and 1240-670 cm^−1^ (amide III), which are consistent with previously reported patterns in the literature [28,29].

The broad band observed at 3327-3419 cm^−1^ corresponds to the stretch –OH groups in collagen. Stretch vibration of C–H were observed at 2970-2997 cm^−1^. The absorption band at 1650 cm^−1^ in Amide I region indicates that stretch vibration of C=O in the polypeptide backbone of the protein. The peaks around 1550 cm^−1^ represent the amide II bands corresponding to N–H bending. The characteristic peaks in the amide A, B and I regions were present in all samples, regardless of the addition of ECM buffer. However, the effect of ECM buffer on collagen and ECM digest was evident in the peaks in the amide II and III regions. Lower intensity absorption bands were observed in collagen and ECM hydrogels (with ECM buffer) compared to collagen and ECM digest at around 1450, 1400, 1340, and 660 cm^−1^. These bands were attributed to CH_2_ bending, CH_2_ wagging of proline, COO- symmetrical stretch, and O=C–N bending, respectively, indicating a greater disorder associated with the loss of the triple helix state of collagen [30]. Peak intensities of around 1080 cm^−1^and 860 cm^−1^, indicative of ether linkage (C–O–C), were increased in the collagen and ECM gels treated with ECM buffer. This observation suggests the presence of NaCl and NaOH in the buffer plays an important role in the gelation of collagen and ECM hydrogels through the formation of the linkage between the polypeptide chains of collagen.

### 3.3 Generation of porous and fibrous hydrogel microstructures

**Fig. 4:**
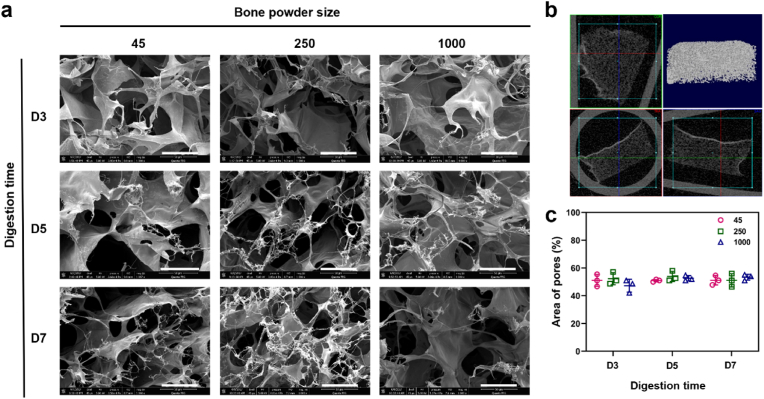
**Microstructure analysis of human bone ECM hydrogels using scanning electron microscope (SEM) and micro-computed tomography (μ-CT).** (a) SEM images of cross-sections of lyophilised ECM hydrogels were observed by SEM (scale bar = 300 μm), showing the presence of porous and fibrous structures in all types of ECM hydrogels. (b) 3-dimensional visualization of the ECM hydrogel structure using μ-CT demonstrates the porous nature of the hydrogels. (c) Measurement of pore sizes in ECM hydrogels using ImageJ software reveals no significant differences between the hydrogels (n = 3). Statistical significance was determined using a two-way ANOVA test with Tukey's multiple comparisons test (∗p < 0.05 ∗∗p < 0.005 ∗∗∗p < 0.0005∗∗∗∗). Data represent mean ± SD, N = 4.

To investigate the effect of bone powder size and digestion time on the microstructure of the gel, we examined the cross-sectional and three-dimensional structures of the hydrogels. All the hydrogels examined exhibited a porous and fibrous structure (Fig. 4a, Supplementary Fig. S2). The fibers were widely distributed throughout the pores, which were observed to be interconnected (Fig. 4a and b). The pore area percentage, calculated based on SEM images using ImageJ, did not display any significant differences between the hydrogels (51.8 ± 4.3 %) (Fig. 4c).

**Fig. 5:**
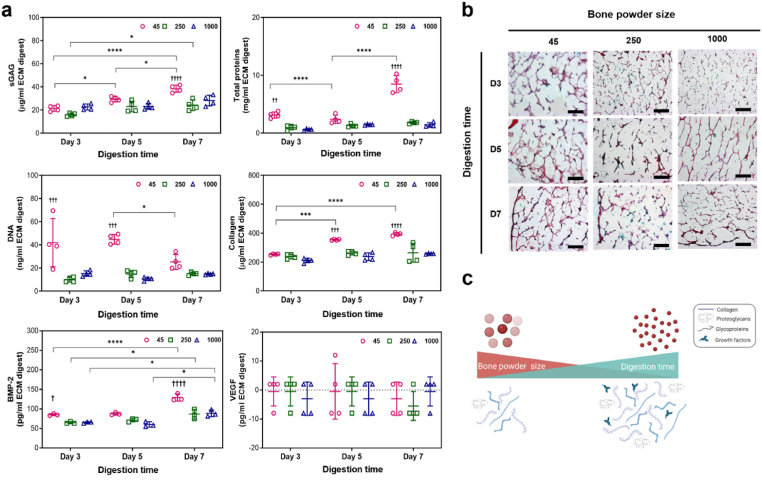
**Concentrations of DNA, total proteins, sGAG, collagen, BMP-2 and VEGF in ECM digests and histological staining images of ECM hydrogels.** (a) Significantly high concentrations of proteins, including sGAG and BMP-2, were detected in the 45–250 μm powder and 7-day digested ECM digest, compared to other ECM digests (n = 4). Statistical significance was determined using a two-way ANOVA test with Tukey's multiple comparisons test (∗p < 0.05 ∗∗p < 0.005 ∗∗∗p < 0.0005∗∗∗∗). The † symbol indicates a significant difference within the groups at the same digestion time). Data represent mean ± SD, N = 4. (b) ECM hydrogels were stained with haematoxylin and Alcian blue/Sirius red (scale bar = 100 μm). (c) Schematic illustration of the effects of bone powder size and digestion time on digesting ECM proteins.

### 3.4 Bone powder size and digestion time affect cellular and protein contents within ECM digests

The concentrations of DNA and proteins present in the ECM digests were quantified. The concentrations of total proteins, sGAG, and collagen significantly increased as bone powder size decreased and digestion time increased (Fig. 5a). Comparison of the ECM digest from 1000 to 2000 μm after 3 days of digestion (referred to as 1000–D3, see Table 1), confirmed that the samples with the smallest bone powder size and the longest digestion time (45–D7) exhibited a higher solubilization of proteins from the ECM, as indicated by the total protein concentrations (8.5 ± 1.5 mg/ml for 45-D7, 0.6 ± 0.16 mg/ml for 1000-D3).

To assess the impact of bone powder size and digestion times on ECM decellularization, demineralized bone powders were separately collected based on their sizes and the powders were digested for 3, 5, and 7 days. The concentration of DNA in smaller bone powder sizes was significantly higher than in larger bone powders. However, with an increase in digestion time, the DNA concentration was decreased. Although further studies are warranted to identify and quantify the DNA content and fragment length, particularly in smaller bone powder sizes, the current studies show that both bone powder size and digestion time are crucial for eliminating cells from the extracellular matrix. The concentration of DNA in 45–D7 ECM digest, obtained from the smallest bone powder with the longest digestion time, was approximately 20 ng/ml (∼2.9 ng of DNA per mg of dried ECM digest), resulting in a composition compatible with other types of ECM hydrogels [31].

To visualize the distribution of proteins within the hydrogels, staining techniques using specific dyes were used. Alcian Blue was used to stain GAGs (blue coloration). Sirius Red for collagen (pink/red coloration) and haematoxylin to stain nuclei (black coloration). The hydrogels exhibited prominent staining for collagen and GAGs (Fig. 5b), while nuclei staining, presenting as a black coloration, was negligible. Consistent with the quantification data (Fig. 5a), ECM hydrogels derived from small bone powder sizes and treated for longer digestion times exhibited stronger staining of collagen and GAGs across larger areas. Furthermore, as depicted in Fig. 4, the staining images revealed the presence of porous structures in all the hydrogels.

In addition to structural protein content, we also quantified the levels of the growth factors BMP-2 and VEGF. BMP-2 was detected in all of the ECM hydrogels with the 45-D7 hydrogel exhibiting the highest concentration of BMP-2 (129 pg/ml BMP-2 ± 9.1, Fig. 5a). However, no VEGF was detected, possibly due to the fact that VEGF is produced by hypertrophic chondrocytes, while BMP-2 is primarily secreted into the bone matrix by pre-existing osteoblasts, osteocytes, and endothelial cells [32,33]. The presence of BMP-2 in the ECM hydrogels indicating their significant potential for application in bone repair applications.

The individual concentrations of proteins, including sGAG, collagen, and BMP-2, indicate that the smallest bone powder size and the longest digestion time enhance the digestion of more proteins from the human bone ECM (Fig. 5a and Supplementary Fig. S3). Interestingly, however, the percentages of sGAG, collagen, and BMP-2 in total protein concentrations increased with the largest bone powder size and the shortest digestion time (Supplementary Fig. S4). The effects of bone powder size and digestion time on protein quantity and quality are closely linked to the role of pepsin solutions in processing human bone powders. Pepsin primarily breaks down proteins into smaller peptides and amino acids. Smaller bone powders are more easily digested by pepsin, leading to more efficient protein breakdown compared to larger powders. However, depending on the digestion time, pepsin can either digest more proteins or break them into smaller peptides. This suggests that the proportions of various ECM proteins in ECM hydrogels depend on the bone powder size and digestion time.

### 3.5 Proteomic analysis of human bone ECM digests

**Fig. 6:**
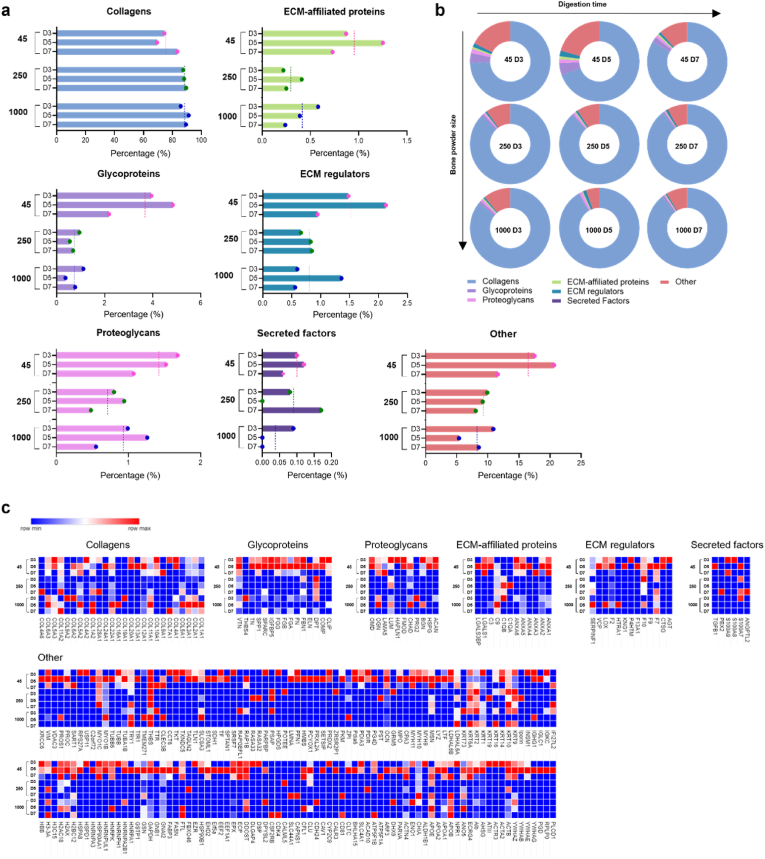
**Comparison of the total matrisome subcategories of proteins in human bone ECM digests.** (a and b) illustrate the percentages of proteins categorized under different matrisome subcategories in ECM digests. ECM digests derived from various sizes of bone powders after 5 days of digestion exhibit a significant impact on the protein proportions compared to those from shorter or longer incubation times. The dashed lines in (a) represent average percentages from the group. (c) The details and proportions of the proteins are listed in the heatmap.

**Fig. 7:**
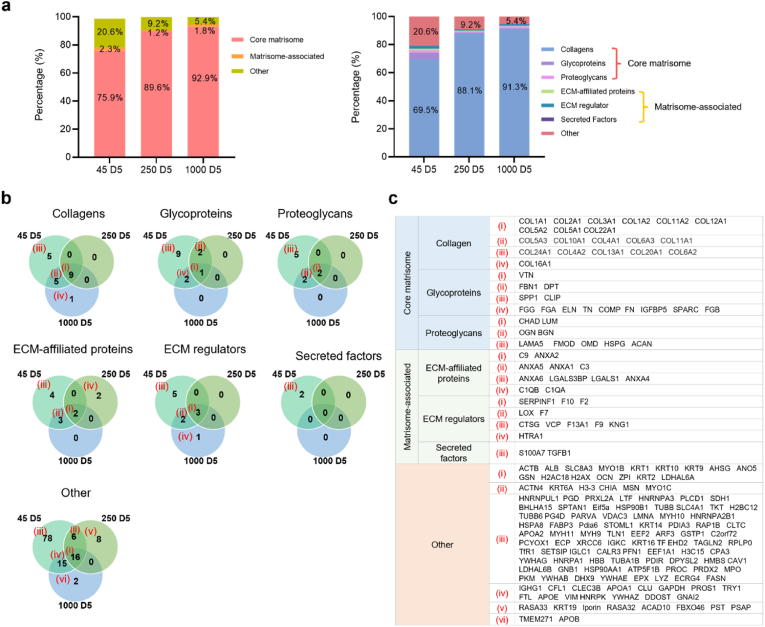
**Total matrisome subcategories of proteins in human bone ECM digests from 45-D5, 250-D5, and 1000-D5.** (a) presents the percentages of categorized proteins from 45-D5, 250-D5 and 1000-D5. (b) shows a Venn diagram depicting the percentage of matrisome subcategories of proteins in various sizes of bone powders after 5 days of digestion. Smaller bone powder exhibits a lower presence of collagens but a higher presence of other protein types. (c) The details of the proteins are listed in the table.

The current studies demonstrated that ECM hydrogels derived from smaller bone size and treated for prolonged digestion times enhance the concentration of proteins from demineralized and decellularized human bone ECM, as indicated by total protein, collagen and sGAG assays (Fig. 5). However, accurately capturing the complex compositions of the ECM remains a challenge. To validate the effect of bone powder size and digestion time on the quality and quantity of ECM, an extensive proteomic analysis of the ECM hydrogels was undertaken (Fig. 6, Fig. 7). Following ECM digestion, a maximum of 246 proteins were identified from the ECM digests, including 52 core-matrisome proteins (collagens, glycoproteins, and proteoglycans) and 31 matrisome-associated proteins (ECM regulators, secreted factors, and ECM affiliated proteins), using the ECM-specific categorization database, MatrisomDB 2.0 [34,35]. The percentage of categorized proteins was calculated based on Score SEQUEST HT, which scores and sums each peptide for a protein.

The impact of bone powder size and digestion time was crucial in determining the proportion of categorized proteins in ECM digests, as demonstrated in Fig. 6a and b. Interestingly, the percentages of all the categorized proteins from ECM digests treated for 5 days were dramatically higher or lower than those treated for 3 days and 7 days, regardless of bone powder size.

Specifically, the ECM digest derived from 45 to 250 μm bone powder size and treated for 5 days (45–D5) demonstrated a lower percentage of collagens but higher percentages of other matrix proteins. Fig. 7 illustrates the percentages of categorized proteins in ECM digests from the different sizes of bone powders after 5 days of incubation (45–D5, 250–D5, and 1000–D5). As bone powder size increased, an observed increase in proportion of core-matrisome proteins (i.e. structural/matrix proteins) was noted and a corresponding decrease in non-matrisome proteins. Thus, within the core-matrisome category, 45–D5 displayed a much lower percentage of collagens (45–D5; 69.5 %, 1000–D5; 91.3 %), but a higher percentage of glycoproteins and proteoglycans were detected (Fig. 7a). Furthermore, a range of proteins categorized as core-matrisome, matrisome-associated, and non-matrisome were identified in 45–D5 ECM digest compared to ECM digests derived from higher bone powder sizes (250–D5 and 1000–D5, Fig. 7b and c). Furthermore, there were more proteins exclusively present in 45–D5 ECM digest (Fig. 6, Fig. 7), including the recognized bone formation-related molecules osteoglycin, osteocalcin, insulin-like growth factor, and transforming growth factor. These findings highlight that smaller bone powders result in the digestion of a higher concentration of proteins with a wider diversity of protein types.

The results of the protein quantification assay (Fig. 5a) clearly demonstrate an increase in protein concentration with decreasing bone size and increasing digestion time. In contrast, proteomic analysis revealed a variety of ECM proteins in smaller bone sizes and moderate digestion times. These disparities can be attributed to differences in the analysis of protein concentrations and the proportions of categorized ECM proteins. However, it is important to note that both sets of results (Fig. 5, Fig. 6) consistently demonstrate the impact of bone powder size and digestion time on promoting both the quantity and quality of ECM proteins.

### 3.6 Human bone ECM hydrogels improve bone marrow-derived stromal cell function

**Fig. 8:**
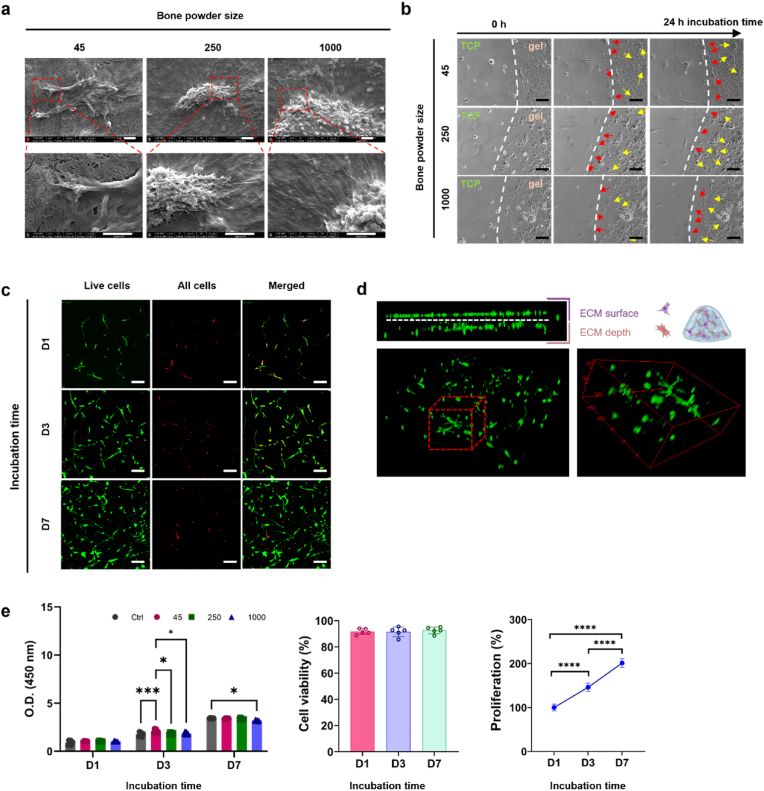
**Behaviour of HBMSCs on human bone ECM hydrogels.** (a) HBMSCs were observed to attach and spread on the ECM hydrogels, irrespective of the hydrogel source from various bone powder sizes (scale bar = 2 μm). (b) ECM hydrogels were placed in the centre of tissue culture plate (TCP), and after seeding HBMSCs, their behaviours were observed using a time-lapse microscope. Some cells from the TCP migrated towards the ECM hydrogels (indicated by red arrows), while cells on the ECM hydrogels spread on top of the hydrogels (indicated by yellow arrows) (scale bar = 50 μm). (c and d) Cells stained with Calcein AM and DiD (green and red, respectively) were cultured in ECM hydrogels for 1, 3, and 7 days. (scale bar = 100 μm) (e) Cell viability and proliferation were evaluated using confocal imaging analysis and the WST-1 assay (n = 5). The cell population was noted to double within 7 days, and there was no significant difference in ECM hydrogels derived from various bone powder sizes. Statistical significance was determined using a one-way test with Tukey's multiple comparisons test (∗p < 0.05 ∗∗p < 0.005 ∗∗∗p < 0.0005∗∗∗∗).(For interpretation of the references to colour in this figure legend, the reader is referred to the web version of this article).

We investigated the ability of human bone ECM to support human bone marrow-derived stromal cells (HBMSCs) given their biological potential in modulating cell response and promoting bone tissue regeneration. Initial studies focused on evaluating HBMSC function, including attachment, spreading, migration viability and proliferation, when cultured on human bone ECM hydrogels derived from various human bone powder sizes and treated for 7 days (45-D7, 250-D7 and 1000-D7). Regardless of the bone powder size examined, HBMSCs exhibited excellent cell attachment and were observed to spread extensively within 30 min of incubation, evidenced by the organization of stress fibers and the formation of filopodia associated with the gel surfaces (Fig. 8a). To compare cell behaviors against control TCP, a droplet of ECM hydrogels was placed in the centre of a tissue culture plate (TCP), and time-lapse recordings performed. (Fig. 8b and videos 1–3). Cells cultured on TCP and the ECM hydrogels exhibited excellent cell spreading and intercellular interactions. Interestingly, examination of cell-movement dynamics revealed chemotaxis of the cells with a preferential migration of cells from TCP towards ECM (Fig. 8b).

Supplementary data related to this article can be found online at https://doi.org/10.1016/j.bioactmat.2024.09.007

The following are the Supplementary data related to this article:

Multimedia component 2 Multimedia component 2

Multimedia component 3 Multimedia component 3

Multimedia component 4 Multimedia component 4

**Fig. 9:**
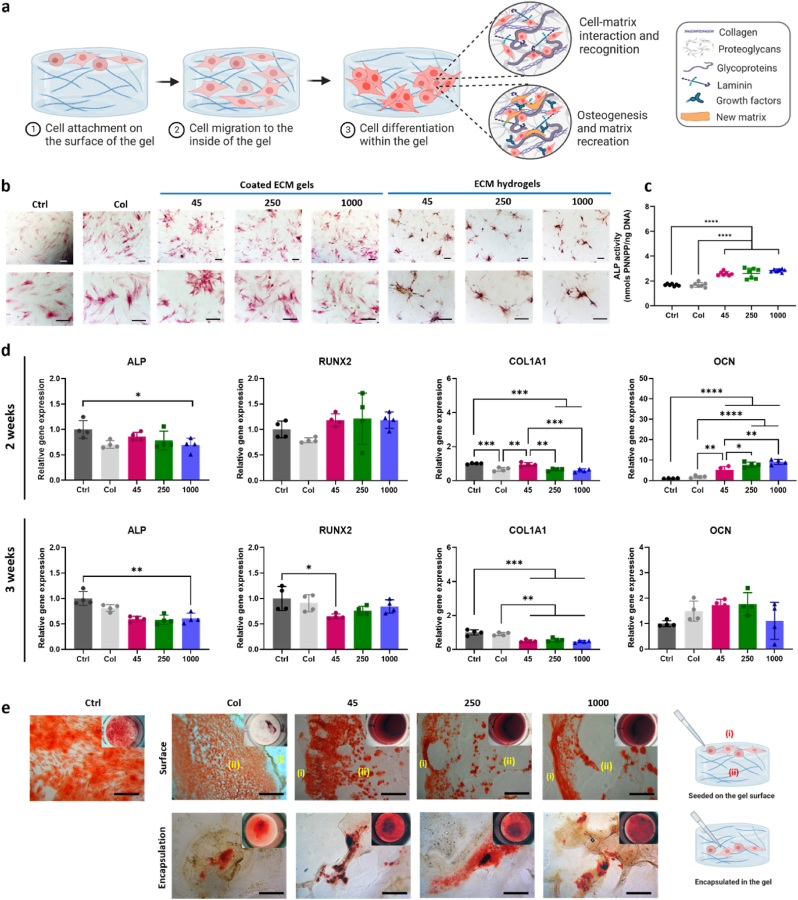
**Impact of human bone ECM hydrogels on HBMSCs osteogenic differentiation and mineralization.** (a) Schematic showing the possible process of cell responses to ECM hydrogels. (b) HBMSCs morphology and alkaline phosphatase (ALP) staining density/colour differed notably between the 2D and 3D settings. (c) After 1 week of culture, ECM hydrogels significantly improved the ALP activities of HBMSCs, compared to tissue culture plate (ctrl) and collagen gels. (d) ECM hydrogels derived from bone powder sizes of 45–250 μm remarkably enhanced the early and late responses of HBMSCs differentiation compared to collagen gels and larger bone powder-sized ECM hydrogels. (e) Alizarin red S staining images of HBMSCs cultured for 3 weeks showed that ECM hydrogels promoted mineralization more effectively than collagen gel (scale bar: 200 μm). Notably, cells encapsulated within the ECM hydrogels exhibited a more robust mineralization response compared to cells seeded on top of the ECM gels. Statistical significance was determined using a one-way test with Tukey's multiple comparisons test (∗p < 0.05 ∗∗p < 0.005 ∗∗∗p < 0.0005∗∗∗∗). Data represent mean ± SD, N = 4.

The presence of a wide variety of proteins in the ECM hydrogels (Fig. 6, Fig. 7) may facilitate cell migration/chemotaxis towards the hydrogels. However, despite significant differences in the release patterns of proteins from the ECM hydrogels (Supplementary Fig. S1), no significant differences in cell migration behavior were observed across the different ECM hydrogels examined. These results suggest that linear boundary of ECM hydrogels stimulated cells to migrate together with an elongated morphology [36,37]. For cells initially seeded on the ECM hydrogels, the porous and fibrous structures of the hydrogels provided an attractive environment for cell exploration (Fig. 4 and Supplementary Fig. S2) [38]. Cell morphology on the ECM hydrogels were distinctly different compared to cells on TCP and ECM digest-coated TCP as observed following staining for alkaline phosphatase expression (Fig. 9b). Cells were observed to invade the porous and fibrous microenvironment of the ECM hydrogels, rather than remaining on the surface. Furthermore, these ECM hydrogel structures promoted cell proliferation, with a doubling in cell numbers within 7 days and excellent cell viability observed (Fig. 8c and e). Although there was no significant effect of varying bone powder sizes of ECM hydrogels on HBMSCs proliferation, the porous and fibrous structures are crucial for recruiting cells to the implanted hydrogels and facilitating their regenerative function within the defects.

Early osteogenic differentiation of HBMSCs was assessed on ECM digest-coated surfaces (2D) and ECM hydrogels (3D) using ALP staining and ALP/DNA assays after 1 week of HBMSCs culture (Fig. 9b and c). Compared to collagen type 1 gel (Col) and control (Ctrl, TCP), ALP staining activities on ECM digest coated surfaces, regardless of ECM digests from different powder sizes, showed a discrete increase in ALP compared to the control and collagen-coated surfaces.

To further assess the osteogenic potential of human bone ECM hydrogels, the gene expression levels of early markers (ALP, RUNX2, and COL1A1) and a late marker (OCN) of osteogenic differentiation were evaluated after culturing HBMSCs on ECM hydrogels for 2 and 3 weeks (Fig. 9d). After 2 weeks, the ECM hydrogels exhibited a slight increase in RUNX2 expression compared to the collagen gel and control group. However, the expression levels of ALP in ECM hydrogels were lower than in the control group. Regarding another early marker, COL1A1, ECM hydrogels derived from 45 to 250 μm sized bone powder (45 ECM) showed significantly enhanced expression compared to the collagen gel and ECM hydrogels from larger bone powder samples. Interestingly, among all the early markers examined, only 45 ECM hydrogels exhibited higher gene expression levels than those from the collagen gel after 2 weeks, but this trend was not observed in the expression levels after 3 weeks. Several studies have shown that ALP, RUNX and COL1A1 activity increase during the first two weeks of osteogenic differentiation and subsequently decline to facilitate mineralization [[39], [40], [41]]. The current observed results suggest the 45 ECM hydrogel has the potential to modulate the early differentiation response of HBMSCs.

After 2 weeks, all the ECM hydrogels exhibited significant upregulation of the late marker OCN gene compared to the control and collagen gel. However, after 3 weeks, there was a notable decrease in OCN expression compared to expression after 2 weeks. Interestingly, the proportion of collagens in the ECM hydrogels, particularly those derived from lower bone powder sizes, was much lower and consisted of various core-matrisome, matrisome-associated, and non-categorized proteins (Supplementary Fig. S5). Collagen Type I has a key role in promoting osteogenic differentiation [42,43]. This suggests that the presence of various proteins in the ECM is crucial for enhancing osteogenic differentiation and the importance of human bone powder size in generating hydrogels consisting of a variety of ECM proteins. Further studies are warranted to evaluate osteogenic differentiation and bone formation capacity of ECM hydrogels, exploring not only variations in bone powder sizes but also digestion times. Such studies would provide valuable insights into identifying key proteins critical for the regenerative potential of the hydrogels.

The current study revealed that cells exhibited a preference for infiltrating into the porous structure of ECM hydrogels rather than remaining on the surface (Fig. 8c and d). To further explore this phenomenon, we compared cell responses between the surface and interior of the ECM hydrogels in terms of mineralization, a hallmark of osteogenic differentiation. To achieve this, we seeded cells on top of ECM hydrogels and encapsulated cells within the ECM hydrogels, followed by Alizarin red S staining after 3 weeks of culture (Fig. 9e).

In comparison to collagen gel, ECM hydrogels promoted a mineralization response, with strong mineralization observed both on the gel surface and within the gel. Conversely, collagen gel did not exhibit this tendency, likely due to gradual degradation, leaving behind a thin layer of collagen gel. When cells were encapsulated within the gels, as opposed to being seeded on the surface, they accumulated within the gels, resulting in a more robust mineralization response compared to cells seeded on top of the ECM hydrogels. Regardless of the cell application method, 45 ECM hydrogels consistently demonstrated a stronger response than 250 and 1000 ECM hydrogels. Further analysis revealed that 45 ECM hydrogels contained higher levels of osteoglycin (OGN) and osteocalcin (OCN) proteins compared to other ECM hydrogels (Fig. 6c). These proteins may have contributed to enhanced osteogenic differentiation and mineralization. Taken together, this study provides compelling evidence for the ability of human bone ECM hydrogels to promote cellular responses for bone tissue regeneration.

## 4 Conclusion

Decellularized ECM hydrogels derived from human bone tissue have garnered significant interest in the field of tissue engineering given their native components and structures. This study represents the successful production of a hydrogel from demineralized and decellularized bone extracellular matrix from human sources. Moreover, we have demonstrated the critical role of bone powder size and digestion duration in the generation of human bone ECM hydrogels and the subsequent modulation of gelation and rheological properties, as well as the quantity and compositional richness of digested proteins within the bone ECM. Notably, smaller bone powder sizes facilitate the digestion of a wider range of ECM proteins offering significant potential as biologically active relevant hydrogels for bone tissue engineering applications.

## Ethics approval and consent to participate

All the tissue samples that would have been discarded were used following informed consent from the patients in accordance with the Southampton & Southwest Hampshire Local Research Ethics Committee (Ref: 194/99/w). All the authors were in compliance with all relevant ethical regulations.

## CRediT authorship contribution statement

**Yang-Hee Kim:** Writing – review & editing, Writing – original draft, Visualization, Validation, Resources, Project administration, Methodology, Investigation, Funding acquisition, Formal analysis, Data curation, Conceptualization. **Gianluca Cidonio:** Writing – review & editing, Writing – original draft, Visualization, Validation, Project administration, Methodology, Formal analysis, Conceptualization. **Janos M. Kanczler:** Writing – review & editing, Writing – original draft. **Richard OC. Oreffo:** Writing – review & editing, Writing – original draft, Funding acquisition. **Jonathan I. Dawson:** Writing – review & editing, Writing – original draft, Project administration, Funding acquisition.

## Declaration of competing interest

The authors declare that they have no competing interests.
